# Oxaliplatin neurotoxicity – no general ion channel surface-charge effect

**DOI:** 10.1186/1477-5751-8-2

**Published:** 2009-01-12

**Authors:** Amir Broomand, Elin Jerremalm, Jeffrey Yachnin, Hans Ehrsson, Fredrik Elinder

**Affiliations:** 1 Department of Clinical and Experimental Medicine, Division of Cell Biology, Linköping University, Linköping, Sweden; 2 Department of Oncology-Pathology, Karolinska Institutet and Karolinska Pharmacy, Karolinska University Hospital, Solna, Stockholm, Sweden; 3 Department of Oncology, University Hospital Uppsala, Uppsala, Sweden

## Abstract

**Background:**

Oxaliplatin is a platinum-based chemotherapeutic drug. Neurotoxicity is the dose-limiting side effect. Previous investigations have reported that acute neurotoxicity could be mediated via voltage-gated ion channels. A possible mechanism for some of the effects is a modification of surface charges around the ion channel, either because of chelation of extracellular Ca^2+^, or because of binding of a charged biotransformation product of oxaliplatin to the channel. To elucidate the molecular mechanism, we investigated the effects of oxaliplatin and its chloride complex [Pt(dach)oxCl]^- ^on the voltage-gated Shaker K channel expressed in *Xenopus *oocytes. The recordings were made with the two-electrode and the cut-open oocyte voltage clamp techniques.

**Conclusion:**

To our surprise, we did not see any effects on the current amplitudes, on the current time courses, or on the voltage dependence of the Shaker wild-type channel. Oxaliplatin is expected to bind to cysteines. Therefore, we explored if there could be a specific effect on single (E418C) and double-cysteine (R362C/F416C) mutated Shaker channels previously shown to be sensitive to cysteine-specific reagents. Neither of these channels were affected by oxaliplatin. The clear lack of effect on the Shaker K channel suggests that oxaliplatin or its monochloro complex has no general surface-charge effect on the channels, as has been suggested before, but rather a specific effect to the channels previously shown to be affected.

## Background

The platinum-based chemotherapeutic drug oxaliplatin has been used in the clinic for about ten years. The therapeutic indication is metastatic colorectal cancer. The mechanism of action is not fully understood, but it is assumed that DNA-adduct formation is one route to cell destruction [1]. The dose-limiting side effect of oxaliplatin treatment is neurotoxicity. A unique and unpleasant acute neurosensory toxicity with paresthesias and dysesthesias of the distal extremities and perioral region occurs shortly after infusion in as much as 90% of the patients. These symptoms can be worsened or triggered by cold, but are reversed within hours or days after treatment. After cumulative doses of about 800 mg/m^2 ^another form of neurotoxicity, with paresthesias and dysesthesias persisting between cycles and problems with sensorimotor coordination is seen in about 10–15% of the patients. Most of the patients recover a few months after treatment discontinuation [2,3]. Many patients who receive a clinical benefit from oxaliplatin cannot continue treatment because of worsening neurotoxicity. Our understanding of the mechanism underlying this is limited and research in this area could lead to prolonged treatment with this useful drug.

Oxaliplatin has a half-life of about 14 minutes *in vivo *and the maximal blood concentration after a 2-hour infusion of 85 mg/m^2 ^is 3.6 μM [4]. The short half-life is explained, at least in part, by the reaction of oxaliplatin with chloride, glutathione, methionine, and cysteine at physiological concentrations [5]. It has been suggested that biotransformation products of oxaliplatin may be responsible for the neurotoxic side effects [6].

Because the symptoms of acute neurotoxicity may be related to hyperexcitability of sensory neurons, the effects of oxaliplatin on voltage-gated Na and K channels, responsible for the shape of the neuronal action potential, have been examined. Oxaliplatin, in the concentration range from 1 to 500 μM, has been shown to affect both Na and K channels [7-11]. Even though these studies on different preparations report on slightly different effects, there seems to be a general theme: The voltage dependence for the Na and K channels is changed (less positive voltages are needed to open the channels), and the Na current inactivation becomes slower and less complete.

Because the voltage dependences of both Na and K channels have been reported to be shifted in negative direction along the voltage axis it is tempting to speculate about a common underlying mechanism. One such possible mechanism is a change of the transmembrane electrical field sensed by the voltage sensor of the ion channels. The voltage sensor is a positively charged α-helix responding to changes in the membrane electric field to open the gate of the ion channel [12]. Metal ions are well known shifters of the channels' voltage dependence along the voltage axis; an increase in the concentration of positive metal ions makes it harder for the positively charged voltage sensor to open the channel and a decrease in the concentration has the opposite effect [13]. Other molecules with shift capacity are polyunsaturated free fatty acids; the negatively charged free fatty acids bind close to the voltage sensor and thereby electrostatically activate the channel by shifting the channels voltage dependence along the voltage axis [14]. Thus, the oxaliplatin effect can be caused either (1) indirectly by chelating extracellular calcium ions by oxalate derived from oxaliplatin, or (2) directly by binding the negatively charged chloride complex of oxaliplatin to the channel. In both cases the positively charged voltage sensor will be electrostatically attracted to the extracellular side, thereby activating the channel. To investigate these hypotheses, we explored the effects of oxaliplatin and its chloride complex on the well-characterized voltage-gated Shaker K channel expressed in *Xenopus *oocytes.

## Results

**Figure 1:**
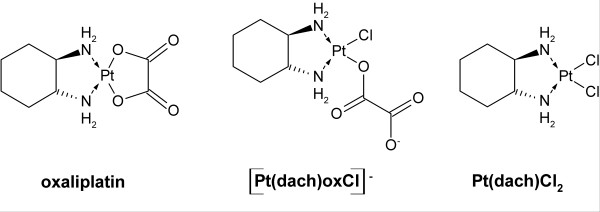
**Chemical structures**. Chemical structures of oxaliplatin, the monochloro monooxalato oxaliplatin complex ([Pt(dach)oxCl]^-^), and the dichloro oxaliplatin complex (Pt(dach)Cl_2_).

Previous investigations have reported effects of oxaliplatin on voltage-gated ion channels. Here, we explored the effect of extracellularly applied oxaliplatin and its monochloro complex ([Pt(dach)oxCl]^-^, Fig. 1) on a well studied voltage-gated K channel, the Shaker K channel expressed in *Xenopus *oocytes.

### Oxaliplatin or [Pt(dach)oxCl]^- ^does not affect the voltage dependence of the wild-type Shaker K channel

**Figure 2:**
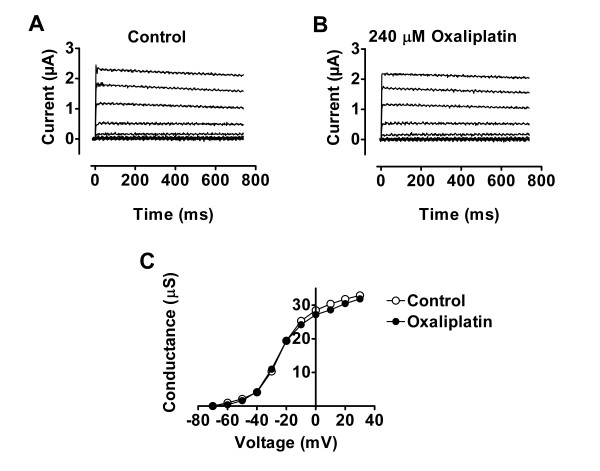
**Shaker wild-type channel**. Oxaliplatin does not affect the Shaker wild-type channel. A) Current family in control solution. Voltage steps between -80 mV and 0 mV in steps of 10 mV. Holding voltage = -80 mV. Time between each step is 2 s. Recordings done by the two-electrode voltage clamp technique. B) Current family with 240 μM oxaliplatin added to the extracellular solution. C) Conductance versus voltage curve for the data from A and B.

We first tested 60 μM oxaliplatin in a Cl^-^-free extracellular solution with the high-resolution cut-open oocyte technique where the oocyte is sandwiched between two holes of two Perspex chambers. The cut-open oocyte technique reduces the capacitive current and thereby allows analysis of the activation time course in detail [15]. The reason for using the Cl^- ^-free solution was to avoid any endogenous Cl^- ^current which could be provoked by the cut-open oocyte technique. No visible effects were seen, neither on the time course, the amplitude, nor on the channel's voltage dependence (*n *= 5; data not shown). One possible explanation for the lack of effect is that it is only [Pt(dach)oxCl]^- ^that exerts effect. Therefore similar experiments with the two-electrode voltage clamp technique were carried out with oxaliplatin added to a Cl^- ^-containing (0.1 M) extracellular solution. Fig. 2A and 2B show a control voltage-clamp family, and a voltage-clamp family with 240 μM oxaliplatin added to the extracellular solution. Fig. 2C shows the corresponding conductance versus voltage, *G*_K_*(V)*, curves calculated as described in Methods. Since most experiments were carried out immediately after the addition of oxaliplatin, a mixture prepared as described in Methods was added to the extracellular fluid to be certain that the oocytes would be exposed to [Pt(dach)oxCl]^-^. Again, the K channels remained unaffected by this treatment. Totally 31 oocytes expressing wild-type channels were investigated with concentrations of oxaliplatin in the range from 60 to 1000 μM. No shift of the *G*_K_*(V) *curve was seen.

For Na channels, it has been reported that the fast inactivation is slowed down considerably [7,8]. To see if this is also applicable to K channels we investigated the effect of oxaliplatin on a fast inactivating K channel (Shaker B). No effect on the inactivation was seen when 60 μM oxaliplatin was added to the extracellular solution (*n *= 2; data not shown).

### Oxaliplatin or [Pt(dach)oxCl]^- ^does not affect a cysteine-mutated Shaker K channel

**Figure 3:**
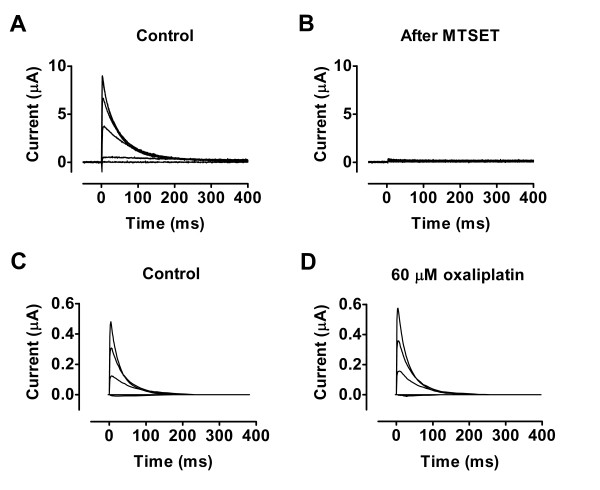
**Cysteine-enriched Shaker channel**. Oxaliplatin does not affect a cysteine-enriched Shaker (E418C) channel. A) Current family in control solution. Voltage steps between -40 and +60 mV in steps of 20 mV. Holding voltage = -80 mV. Time between each step is 2 min to allow complete recovery after inactivation [16]. Two-electrode voltage clamp in A and B. B) Current family after complete modification of the positively charged MTSET. This shows that 418C is very sensitive to modification. C) Another control recording similar to that in A. Cut-open oocyte voltage clamp in C and D. D) Addition of 60 μM oxaliplatin does not affect the currents.

Oxaliplatin is expected to bind to cysteines [5]. Therefore, another explanation for the lack of effects of oxaliplatin is that a critically exposed cysteine residue is lacking in the wild-type channel. To explore this we used the 418C mutation which has a very critical cysteine residue exposed to the extracellular solution. In a previous study we showed that modification of this residue had a large effect on the gating [16]. Fig. 3A shows a voltage-clamp family of 418C currents and Fig. 3B shows that the current is almost completely abolished after modification with the cysteine-specific reagent MTSET. Wild-type Shaker K channels are not affected by MTSET (data not shown). Again, addition of oxaliplatin failed to affect the currents (Fig. 3C and 3D), suggesting that no binding to or modification of 418C occurs (*n *= 8).

### Oxaliplatin or [Pt(dach)oxCl]^- ^does not affect a double cysteine-mutated Shaker K channel

**Figure 4:**
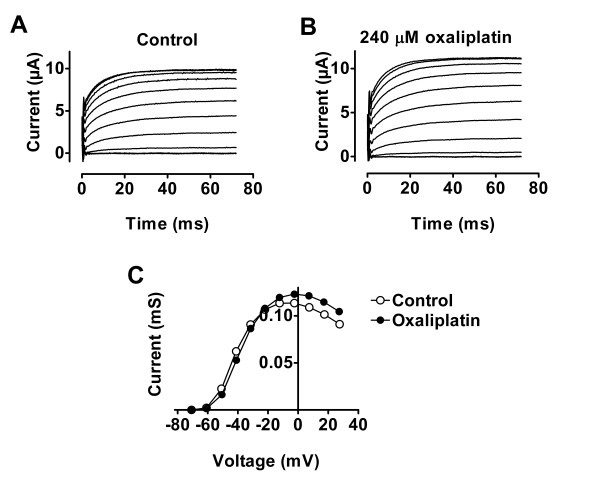
**Double-cysteine-mutated Shaker channel**. Oxaliplatin does not affect a double-cysteine-mutated Shaker (R362C/F416C) channel. A) Current family in control solution. Voltage steps between -80 and +30 mV in steps of 10 mV. Holding voltage = -80 mV. Time between each step is 2 s. B) Current family with 240 μM oxaliplatin added to the extracellular solution. C) Conductance versus voltage curve for the data from A and B. The experiments were carried out in Cl^- ^-containing solutions with the two-electrode voltage clamp technique.

Some substances, like Cd^2+^, have a much larger effect on two neighbouring cysteines than on a single cysteine [17]. In this case Cd^2+ ^makes a metal ion bridge between the two residues. We investigated if a double cysteine mutation (362C/416C), with the cysteines close enough to each other to cause a disulfide bond [18], could coordinate with a platinum complex. Once again, we could not detect any such effects (Fig. 4) (*n *= 5).

## Discussion

In the present investigation we showed that oxaliplatin and its chloride complex [Pt(dach)oxCl]^- ^did not have any effects on the Shaker K channel, neither on wild-type (both inactivating and non-inactivating) nor on cysteine-mutated channels (418C, 362C/416C). This suggests that the neurotoxic side effects reported for oxaliplatin cannot be explained with general effects on the channels' voltage dependences.

### Neurotoxicity of oxaliplatin and its degradation products

Oxaliplatin therapy gives rise to two distinct types of neurotoxicity. One appears after relatively high cumulative doses (10–15% incidence at 800 mg/m^2^) [2] and this side effect seems to be comparable to the neurotoxicity seen after cisplatin and ormaplatin use and has been shown to affect dorsal root ganglion neurons [6,19-22]. The other type of neurotoxicity is an acute form that appears during or within hours or days after administration [3].

The reaction of oxaliplatin with chloride has previously been unravelled [23]. A ring-opened intermediate, the monochloro-monooxalato complex (Fig. 1) is rapidly formed (within 30 minutes) in physiologic *in vitro *conditions, constituting about 10% of the intact oxaliplatin. We hypothesized that this reaction product, which is negatively charged, might be responsible for the acute neurotoxic side effects of oxaliplatin treatment. As mentioned in the Introduction, adding positively charged metal ions shift the channel's voltage dependence in positive direction along the voltage axis [13], while negatively charged compounds shift the voltage dependence in negative direction [14]. Similar electrostatic effects have also been demonstrated for covalent modification of cysteines with reagents of different charge [24].

We know that oxaliplatin is highly reactive towards sulphur containing compounds [5]. The half-life of oxaliplatin in the presence of 2.5 mM cysteine is for example less than 20 minutes. Some K channel mutations used in the present investigation have target sulphur-containing amino acids (cysteine) at the surface. In spite of this, no measurable effect of oxaliplatin on these ion channel subtypes was recorded. This surprising lack of effect may suggest that the cysteines on the channels surface does not react with oxaliplatin, despite high reactivity with other cysteine-specific reagents [18,24]. This also suggests that it is unlikely that oxaliplatin-induced side effect is caused by any covalent modification of channel cysteines.

### Comparison with previous electrophysiological studies

Several previous investigations have reported acute effects on voltage-gated ion channels, and the oxaliplatin-induced side effects have been suggested to be caused by effects on these voltage-gated ion channels [7-11]. Four of these studies report on positive Na channel effects and two on K channel effects. However, in most of these investigations, very high concentrations of oxaliplatin were used. The maximum blood concentration of intact oxaliplatin after a 2-hour infusion of 85 mg/m^2 ^is 3.6 μM [4]. Three of the studies only used high concentrations of 25–500 μM [7-9], while two of the studies (both of which reported on K channel effects) used more physiologically relevant concentrations down to 1 μM [10,11].

General mechanisms have been suggested. 1) Oxalate liberated from oxaliplatin could exert general effects by chelating Ca^2+ ^and thereby affecting fixed surface charges [7,10]. The present study does not support such a mechanism, since no effects were seen in the ion channel setting in spite of different amounts of oxalate released from oxaliplatin using varying exposure times (5 min-12 h) and concentrations (60–1000 μM). Furthermore, it is difficult to understand how the relatively small *in vivo *concentrations of oxalate could be sufficient to deplete the extracellular calcium pool of about 1 mM. Moreover, since the intracellular Ca^2+ ^concentration is in the μM range, formation of calcium oxalate intracellularly, as suggested by Grolleau et al [7], as a precipitate or an ion pair is highly unlikely [25]. Intracellular Ca^2+ ^has been reported not to be affected by extracellular oxaliplatin [9]. 2) Another possibility is that the negatively charged monochloro-monooxalato complex (Fig. 1) exerts general effects by binding directly to ion channels (one of the hypotheses put forward in the present investigation). Neither this is supported in the present investigation.

A negative shift in the voltage dependence of Na-channel activation that could be responsible for the hyperexcitability seen in the acute oxaliplatin side effects was detected in some investigations [8,10]. A slowing of the Na-channel inactivation could also promote hyperexcitability. In some Na channels, such a slowing has been reported [8,9], while it is lacking in other Na channels [8,10]. All together, this points to channel-specific effects rather than general effects.

## Conclusion

Oxaliplatin at relatively high concentrations has been reported to affect the voltage dependence of some ion channels. Here, we have shown that these reported effects are not unspecific channel effects and that these effects are probably not mediated via binding to cysteine amino acid residues. Instead, we propose that the side effects of oxaliplatin are caused by specific channel effects, most likely a slowing of inactivation in some Na channels.

## Methods

### Molecular biology and expression of ion channels

The experiments were performed on the Shaker H4 potassium channel [26], made incapable of fast-inactivation by the Δ6–46 deletion [27], and on the fast inactivating Shaker B channel [28]. For the experiments with cysteine-mutated Shaker H4 channels we used the mutations E418C [16] and R362C/F416C [18]. cRNA was transcribed using the T7 mMessage mMachine kit (Ambion Inc., Austin, TX, USA) and injected in *Xenopus laevis *oocytes (20–500 pg/cell) using a Nanoject injector (Drummond Scientific Co., Broomall, PA, USA). The oocytes were maintained in a modified Barth's solution (MBS, in mM: 88 NaCl, 1 KCl, 2.4 NaHCO_3_, 15 HEPES, 0.33 Ca(NO_3_)_2_, 0.41 CaCl_2_, and 0.82 MgSO_4_) adjusted to pH 7.5 by NaOH, and supplemented with penicillin (10 μg/ml), streptomycin (10 μg/ml) and sodium pyruvate (10 μg/ml). The electrophysiological experiments were made 3–5 days after injection of mRNA.

### Electrophysiology and analysis

Totally, 50 oocytes expressing inactivating, non-inactivation, or cysteine-mutated Shaker K channels were investigated with different concentrations of the platinum drug. The currents were measured with the two-electrode voltage clamp technique or the cut-open oocyte voltage clamp technique (CA-1 amplifier, Dagan Corporation, Minneapolis, MN, USA) as previously described [15,16,29]. Microelectrodes were made from borosilicate glass and filled with a 3 M KCl solution. The resulting resistance varied between 0.5 and 2.0 MΩ. For the two-electrode recordings we used the 1 K solution (in mM: 88 NaCl, 15 HEPES, 1 KCl, 0.8 MgCl_2_, and 0.4 CaCl_2_) adjusted to pH 7.4 by NaOH. For the cut-open oocyte recordings, the solutions in the (extracellular) top pool and the guard pool were composed of (in mM): 107 KOH, 107 methanesulfonic acid, 10 HEPES, and 2 CaCl_2_. The solution in the (intracellular) lower pool was (in mM): 110 KOH, 110 methanesulfonic acid, 10 HEPES, and 0.1 EGTA. The amplifier's capacitance and leak compensation were used, and the currents were low pass filtered at 5 kHz. All experiments were carried out at room temperature (20–23°C).

To covalently modify a substituted cysteine, the membrane-impermeant thiol reagent MTSET ([2-(trimethylammonium)ethyl]methanethiosulfonate bromide) (Toronto Research Chemicals Inc., North York, Ontario, Canada), was applied continuously in the bath solution by a gravity-driven perfusion system. The cysteine reagent was applied to saturation (1 mM for 1 minute) followed by carefully washing off MTSET.

The steady-state K conductance *G*_K_*(V) *was calculated as *G*_K_(*V*) = *I*_K_(*V*)/(*V *- *E*_K_), where *I*_K_*(V) *is the steady-state current,*V *is the membrane potential measured in the bulk solutions, and *E*_K _is the equilibrium potential (-80 mV).

To make sure that the platinum-based substances reach the ion channels and do not get stuck in the vitelline layer surrounding the *Xenopus *oocytes, we tested the effects on oocytes with the vitelline layer mechanically removed. No difference was seen between the recordings with or without the vitelline layer (*n *= 3). Because removal of the vitelline layer makes the oocytes fragile we kept it intact throughout the rest of the experiments.

### Oxaliplatin chemistry and oocyte exposure

Oxaliplatin (Fig. 1) was purchased from Sigma-Aldrich and dissolved in MilliQ water by ultrasonification for 1 hour resulting in a concentration of 6 mM. Oxaliplatin was then added to the extracellular solution, resulting in a concentration of typically 60, 120, or 240 μM. In most cases we measured the effects directly after oxaliplatin exposure but we also tried application times up to 1 hour. Some experiments were also performed with over-night exposure. For these experiments no control recording could be obtained, making it difficult to explore subtle effects on the ion channel kinetics. To produce the monochloro monooxalato complex ([Pt(dach)oxCl]^-^, Fig. 1) NaCl was added to the oxaliplatin solution to final concentrations of 0.2–0.3 M and 1–3 mM, respectively and heated for about 20 minutes at 37°C and then quenched on ice. This gives a mixture of oxaliplatin, Pt(dach)Cl_2 _(Fig. 1, trace amounts), and [Pt(dach)oxCl]^- ^(about 10%) [23] This mixture was added to the extracellular solution corresponding to a platinum concentration of 60, 120, or 240 μM.

## Competing interests

The authors declare that they have no competing interests.

## Authors' contributions

AB carried out the electrophysiological experiments and drafted the manuscript. EJ prepared the platinum compounds and drafted the manuscript. JY initiated the study and helped to draft the manuscript. HE participated in the design of the study and helped to draft the manuscript. FE conceived of the study, and participated in its design and coordination and helped to draft the manuscript.
